# Light Chain Stabilization: A Therapeutic Approach to Ameliorate AL Amyloidosis

**DOI:** 10.3390/hemato2040042

**Published:** 2021-10-05

**Authors:** Gareth J. Morgan, Joel N. Buxbaum, Jeffery W. Kelly

**Affiliations:** 1 Section of Hematology and Medical Oncology, Department of Medicine, Boston University School of Medicine, Boston, MA 02118, USA; 2 The Amyloidosis Center, Boston University School of Medicine, Boston, MA 02118, USA; 3 Department of Molecular Medicine, The Scripps Research Institute, La Jolla, CA 92037, USA; 4 Department of Chemistry, The Scripps Research Institute, La Jolla, CA 92037, USA; 5 The Skaggs Institute for Chemical Biology, The Scripps Research Institute, La Jolla, CA 92037, USA

**Keywords:** light chain amyloidosis, amyloid fibrils, antibody light chains, drug design, kinetic stabilizer, cardiomyopathy, pharmacological chaperone

## Abstract

Non-native immunoglobulin light chain conformations, including aggregates, appear to cause light chain amyloidosis pathology. Despite significant progress in pharmacological eradication of the neoplastic plasma cells that secrete these light chains, in many patients impaired organ function remains. The impairment is apparently due to a subset of resistant plasma cells that continue to secrete misfolding-prone light chains. These light chains are susceptible to the proteolytic cleavage that may enable light chain aggregation. We propose that small molecules that preferentially bind to the natively folded state of full-length light chains could act as pharmacological kinetic stabilizers, protecting light chains against unfolding, proteolysis and aggregation. Although the sequence of the pathological light chain is unique to each patient, fortunately light chains have highly conserved residues that form binding sites for small molecule kinetic stabilizers. We envision that such stabilizers could complement existing and emerging therapies to benefit light chain amyloidosis patients.

## 1. Introduction

It has long been known that immunoglobulin light chain (LC) amyloidosis (AL) is associated with either a highly proliferative plasma cell neoplasia, or much more limited clonal expansions in which the major manifestation of the disease is organ compromise caused by some combination of LC misfolding, misassembly and deposition of amyloid fibrils [1]. George Glenner’s studies of the amino acid sequence of fibrils extracted from the tissues of either multiple myeloma patients with amyloidosis or from patients with “primary” or AL amyloidosis revealed sequence homology or identity with the patients’ circulating or urinary LCs [2–4]. This was the first identification of any human systemic amyloid precursor protein. From the initial description of LCs as the component of amyloid fibrils in AL, aberrant proteolysis was suspected to play a role in fibrillogenesis [5]. A comprehensive review of LC related proteins found in AL tissue deposits revealed that LC fragments were universally present, frequently found in combination with intact full-length LCs [6]. Full-length (FL) LCs were rarely the sole component of the deposit. The identified fragments contained either variable (V) regions, partial V-regions, V-regions associated with a portion of the constant (C) region or combinations thereof [7–9]. Some, but not all, urine-derived LCs (known as Bence-Jones proteins, or BJPs) could be induced to form amyloid-like fibrils by proteolysis [5,10]. The relationship between amyloid pathology and amyloid fibril formation in vitro was, and remains, unclear since not all LCs from AL patients formed amyloid following proteolysis in vitro, while some LCs from individuals without amyloid deposits readily aggregated after proteolysis.

The hypothesis that plasma cell proliferation, resulting in increased secretion of misfolding-prone FL LCs, was responsible for AL, directed therapy toward eradicating the expanded clone. A regimen including melphalan and prednisone, at that time the preferred therapy for multiple myeloma, was the initial cytotoxic choice [11]. It was assumed that elimination of the plasma cell clone would enable amyloid clearance by reducing or abolishing the source of FL LC that could misfold with aberrant proteolysis and add to amyloid deposits by templating or seeding. However, in the early days of clone eradication therapy for cardiac AL, it was noted that a positive functional cardiac response was much more rapid than would have been expected if the primary mechanism involved clearance of amyloid deposits from tissue [12]. Further, in patients showing a clinical cardiac response, the amyloid fibril load seemed unchanged. A similar observation was recently made with kinetic stabilizer treatment of transthyretin (TTR) cardiomyopathy [13]. Collectively, these clinical results suggested that reducing the concentration of secreted FL LCs lowers the soluble non-native LC load, including the concentration of circulating aggregates, abrogating their direct toxic effects now known to be mediated through the p38α MAPK pathway [14–19]. Both actively growing amyloid deposits and circulating soluble, non-native LCs appear to play a pathogenic role in AL. Amyloid deposits that have been in the tissue for extended periods are associated with serum amyloid *P* component, extracellular matrix components such as glycosaminoglycans, and lipids and lipoproteins which appear to kinetically stabilize the fibrils [20–23]. Freshly deposited amyloid with fewer stabilizing accessory macromolecules may be more prone to dissociate and contribute to the putatively toxic soluble circulating non-native LC pool.

It appears that not all LCs have the capacity to form amyloid in vivo, but presumably can still misfold, misassemble and deposit in a non-fibrillar form, as seen in monoclonal immunoglobulin deposition disease (MIDD) [24]. Individuals with multiple myeloma often have much higher levels of circulating FL LC than those with AL (median involved free LC 494 mg/L in myeloma vs. 178 mg/L in AL) [25,26], presumably due to the higher numbers of clonal LC producing plasma cells in myeloma and more rapid clearance of circulating LC due to tissue deposition in AL. While LC amyloid is seen in approximately 10–30% of patients with myeloma [27], the most common form of LC tissue dysfunction in myeloma is the kidney disease related to the non-amyloid aggregates that form renal tubular casts [28]. LCs derived from individuals with multiple myeloma have been shown to be less toxic in cells and animal models than LCs from AL patients [14,29,30].

We hypothesize that AL-associated LCs exhibit an increased propensity to form soluble non-native LC structures that are toxic to organs. From this perspective, the ubiquitous proteolysis of LCs observed in AL amyloid fibrils is a consequence of their tendency to form non-native structures, which are likely to be more protease-labile than natively folded LCs [31]. Circulating non-native LC structures can also misassemble into amyloid fibrils, with or without proteolysis, hence more than one peptide sequence derived from the same AL LC may be present in amyloid fibrils. In other amyloid diseases, soluble non-native oligomers formed from the precursor proteins are hypothesized to be toxic [32–34]. Oligomers of LCs or their proteolytic fragments are likely intermediates on the way to fibril formation, but there is little evidence for their presence in patients because current methods used to detect them are not conformation specific.

Even now, with the availability of multiple, highly potent anti-plasma cell proliferative regimens (therapeutic strategies capable of achieving rapid, deep and durable hematologic responses, HR), the proportion of patients not achieving a full organ response is significant (Table 1) [25,35–38]. In contrast to myeloma, even the presence of low levels of therapy-evasive AL-associated plasma cells secreting FL LCs may lead to worsening of symptoms, because these FL LCs are unstable and more readily convert to toxic non-native LCs. Heretofore the therapeutic response to progression or recurrence of AL after treatment has been the institution of a new cytotoxic regimen, even though the available treatments are significantly toxic and the development of resistance is common [39].

Complete eradication of the plasma cell clone appears to be possible in some patients, especially for those who can tolerate intensive therapies [45,46]. Multiple studies have shown that deeper hematological responses predict improved organ responses and survival [25]. However, for most patients, it appears that reduction in the pathogenic FL LCs below current limits of detection may not be sufficient for long-term remission. Free light chain assays measure changes in the ratio of κ to λ LCs, since absolute levels can vary by several orders of magnitude [47]. Once the monoclonal LC levels are reduced to within the healthy range, this ratio becomes more difficult to measure accurately and to interpret. Absolute levels of κ or λ LCs measured by standard free light chain immunoassays or mass spectrometry appear to provide better prognostic information than free LC ratios [48–50]. Quantifying the specific clonal LC by mass spectrometry is particularly promising for AL, where only a fraction of the total LC may be pathogenic. The recently introduced MASS-FIX method has a higher sensitivity and specificity than previous assays but requires specialist instrumentation that is not yet widely available [50,51]. New, easily measured and interpreted biomarkers are needed to provide more accurate prognoses.

A complementary method for gauging response to therapy involves measuring levels of clonal plasma cells from a bone marrow biopsy to evaluate “minimal residual disease” (MRD) using flow cytometry or deep sequencing of antibody genes [52]. MRD positivity is typically defined as the presence of more than one clonal cell per 10^5^ or 10^6^ cells in bone marrow. Although MRD measurements have potential as useful biomarkers, the relationships between MRD, free LC levels and organ responses are not yet clear [53–56].

While there is significant debate as to which biomarkers best predict a patient’s prognosis, patients whose “involved free LC” levels are most effectively suppressed by therapy have the best prognosis in terms of both organ response and overall survival [48,49]. Studies showing complete hematologic responses with ongoing organ pathology, i.e., HR without organ response, perhaps should be viewed with some skepticism until better methods are developed to specifically detect low levels of circulating non-native LCs.

**Figure 1.**
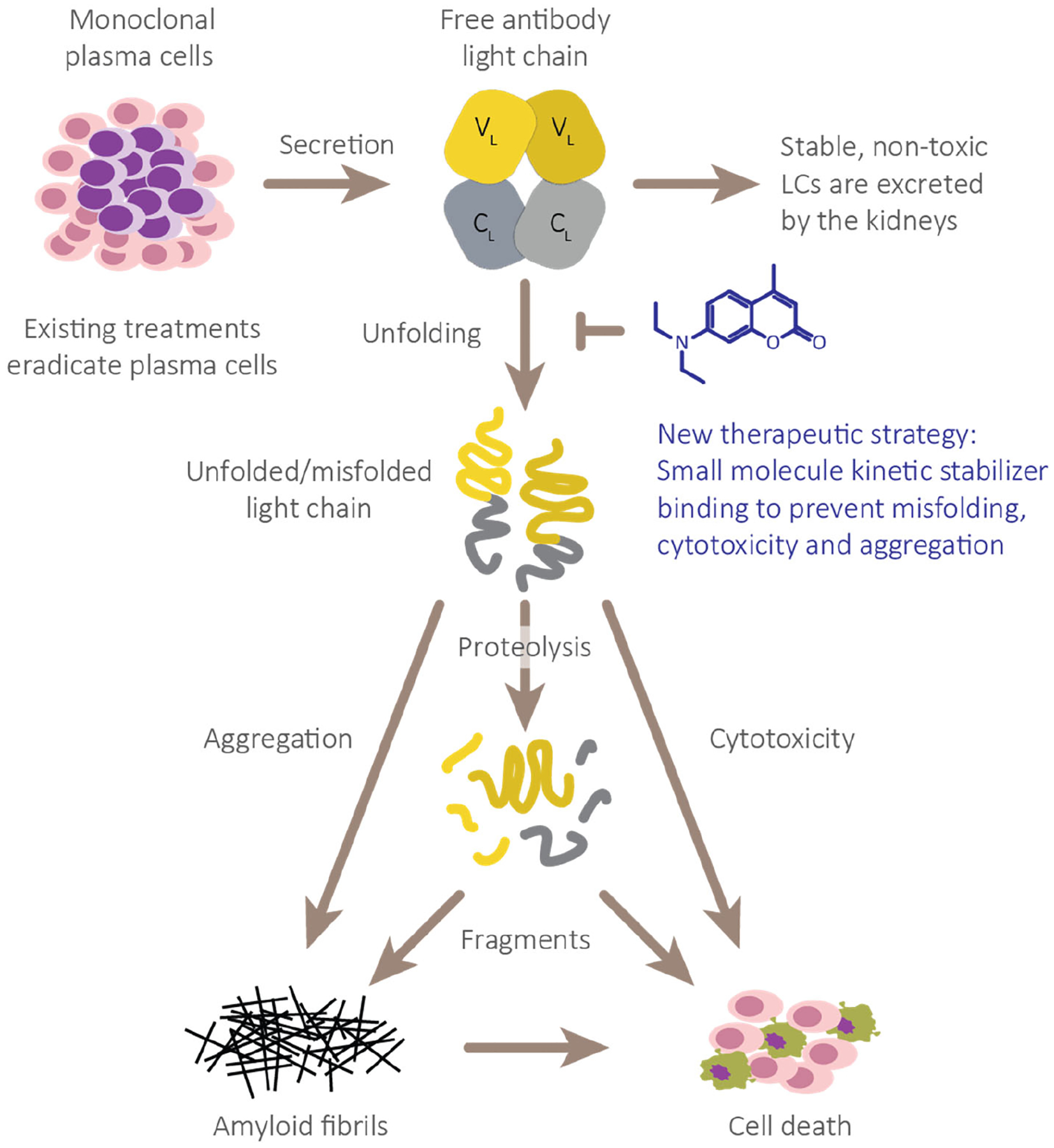
Full-length immunoglobulin LC aggregation cascade thought to cause AL amyloidosis pathology. Full-length immunoglobulin LC dimers secreted from clonal plasma cells normally remain folded and are removed by glomerular filtration and tubular reabsorption and degradation. However, unstable LCs exhibit an increased propensity to adopt non-native, proteolytically labile conformations. These species or their proteolytic fragments are predisposed to aggregate into a variety of non-native structures including amyloid fibrils, which collectively destroy tissues that do not fully functionally recover. We propose that stabilization of the native LC dimers will prevent their unfolding, thereby suppressing formation of all pathological non-native species.

The pathological LC can misfold or misfold and misassemble into a spectrum of non-native structures, with or without aberrant proteolysis (Figure 1) [57–59]. Therefore, we and others have posited that a conservative therapeutic strategy would be to inhibit the entire process of misfolding, aberrant endoproteolysis and misassembly of LCs by stabilizing the native fold of FL LCs using a small molecule that binds to the LC native state [60–62]. Notably, the FL LC small molecule stabilizer strategy should suppress proteotoxicity originating from circulating soluble non-native LCs in cases of incomplete elimination of clonal plasma cells. A very safe small molecule FL LC binder and stabilizer is being sought, because such a molecule could be used as an adjuvant to a plasma cell cytotoxic regimen or used alone as long-term maintenance therapy.

The pathological FL LCs being secreted by proliferating plasma cell clones are highly diverse, having undergone affinity maturation and selection for antigen binding [63,64]. Mature FL LC sequences are derived from one of many precursor gene fragments in either the κ or λ locus, and further modified by clone-specific mutation. Therefore, each patient has a unique FL LC protein sequence conferring a range of properties, including their propensity to form dimers. Dimerization of native LCs appears to reduce their propensity to misfold and aggregate, and further homodimer stabilization by formation of an inter-chain disulfide bond may further suppress aggregation [58,65]. Multiple post-translational modifications have been observed on FL LCs isolated from patients, but the roles of these modifications in pathology are not understood. Notably, a subset of AL-associated LCs is more likely than other LCs to be *N*-glycosylated [66–68]. It is not clear how glycosylation, which can often stabilize proteins and lower their aggregation propensity, is related to amyloidosis.

If every AL patient has a unique light chain sequence, how could a single small molecule FL LC stabilizer be useful in most patients? The sequence diversity of FL LCs is concentrated in the antigen-binding loops known as complementarity-determining regions. The residues that form the interface between heavy chains and LCs are highly conserved to allow efficient antibody heterodimerization. These residues are conserved between the germline precursor gene sequences and between both amyloidogenic and non-amyloidogenic LCs. We discovered that our small molecule stabilizers bind to these highly conserved amino acid residues [60]. Since a key determinant of pathology appears to be the ability of a given FL LC, or its V-region released by proteolysis, to form non-native conformations, stabilizing the native FL LC pharmacologically blocks the conformational excursions required for misfolding, or misfolding, aberrant proteolysis and misassembly, processes leading to organ toxicity. Although we do not know the exact non-native structures leading to organ toxicity in AL, we hypothesize that suppressing formation of all these non-native structures by stabilizing the natively folded state of the FL LC should substantially reduce FL LC misfolding, aberrant proteolysis, and aggregation–processes leading to organ toxicity.

## 2. AL Amyloid Is Assembled from Non-Natively Folded LC Peptides

**Figure 2.**
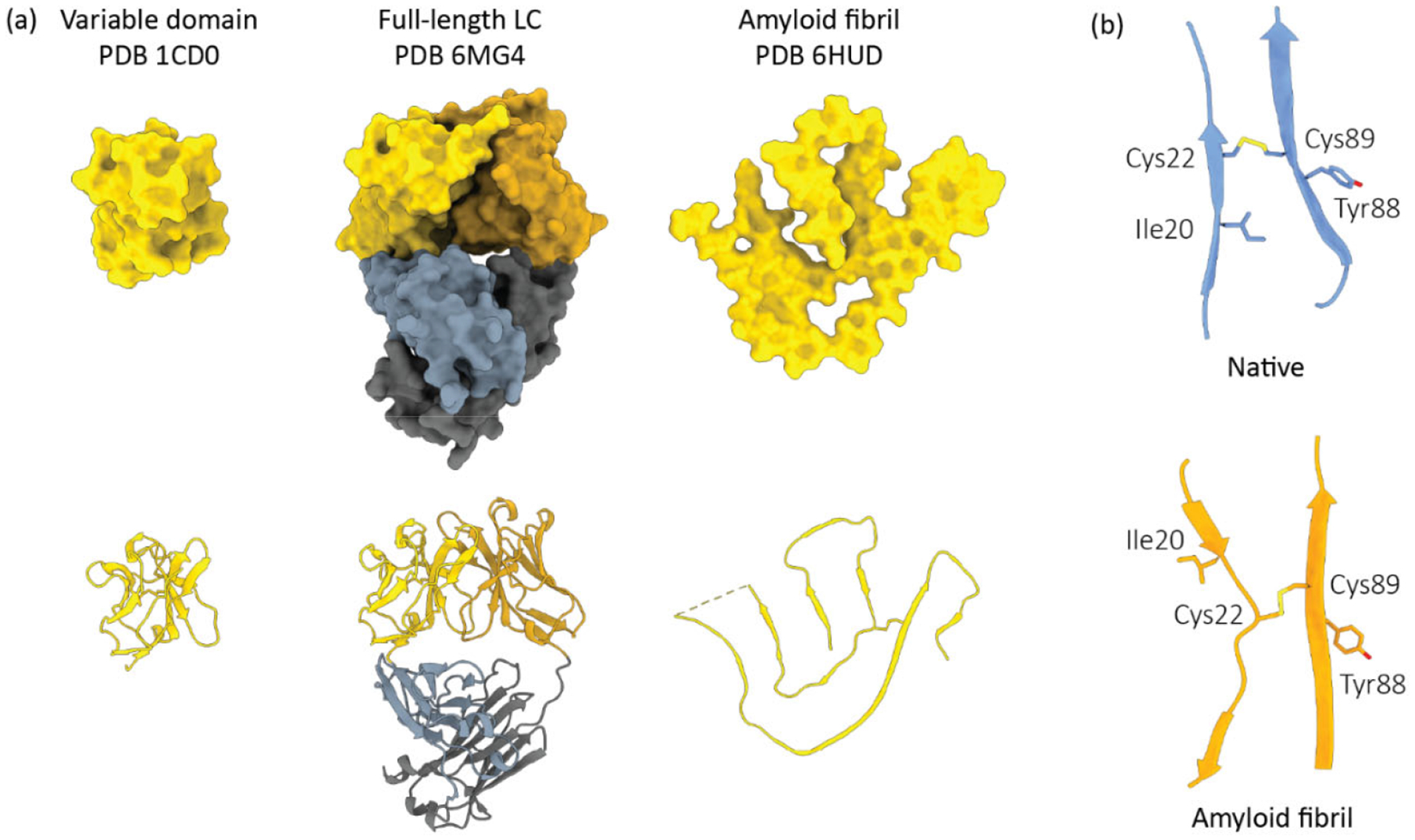
Light chain conformational changes associated with AL amyloid formation. (**a**) The structures of LC variable domains, full-length LC dimers and an amyloid fibril are shown from left to right. All LCs are derived from the *IGLV6–57* precursor gene, which is over-represented in AL. LC species are depicted as solvent-accessible surfaces (top) and ribbon diagrams (bottom). (**b**) The peptide chain orientation around the conserved disulfide bond between LC variable domain residues 22 and 89 (Kabat numbering).

Fifty years after Glenner’s discovery that LCs are amyloidogenic proteins, cryo-electron microscopy-derived atomic resolution structures of AL amyloid fibrils purified from patients have become available. The structured cores of fibrils taken from three individual patients are formed by the V-region of the LC, with an apparent proteolytic cleavage removing some or all the C-region (Figure 2) [69–71]. Similar fragmentation of the LC precursor has been observed in fibril deposits by mass spectrometry [21]. These studies support the earlier work highlighting a key role for proteolysis in AL.

Seeking to understand the role of FL LCs in pathology, we compared aggregation of FL LCs with their constituent V-regions [58]. FL λ LC dimers, which are stabilized by an inter-molecular disulfide bond, are highly resistant to aggregation under conditions where the isolated V-regions readily form aggregates, including amyloid fibrils. Destabilization of the dimeric structure leads to an increased propensity to aggregate [65]. Similarly, FL κ LCs aggregate much more slowly than their associated V-regions [72]. These data are consistent with the structural constant domain acting as a “self-chaperone” that protects the LC from aggregation, supporting the hypothesis that proteolysis by unidentified proteases is involved in initiating LC aggregation.

High resolution mapping of peptide termini by mass spectrometry recently revealed a more complex picture, consistent with multiple proteolytic events after amyloid fibril formation [73,74]. Proteolytic events after fibril formation are also suggested by the recent cryo-electron microscopy-delineated structures of TTR amyloid fibrils in the hearts of late-onset familial amyloidosis patients [75]. These observations are not necessarily contradictory since multiple species derived from the same LC are clearly present in deposits. One possible model is that proteolysis of misfolded precursor proteins yields amyloidogenic fragments that are responsible for initiation of amyloid deposition, after which FL LCs could be deposited by a seeding process with further proteolysis after deposition. In support of this model, fibrils formed from V-regions were shown to accelerate aggregation of full-length LCs [76], a process that should be slowed by small molecule FL LC stabilizer binding to the native state.

The high-resolution structures clearly show that LC amyloid fibrils are non-native protein assemblies that cannot be built directly from natively folded LCs (Figure 2). Substantial unfolding and reorganization of the peptide chain is required for amyloid formation. Notably, the intramolecular disulfide bond between cysteine residues 22 and 89 (numbered according to the Kabat system [77]) of the V-region is intact in all amyloid fibril structures reported thus far. Reduction of this bond is not required for amyloidogenesis in vitro, but the orientation of the peptide chain around the disulfide bond is reversed in the fibril compared to the native state (Figure 2b), consistent with a highly unfolded misassembly transition state [69].

## 3. (Kinetic) Stability as a Measurable, Unifying Parameter to Define LC Toxicity

**Figure 3.**
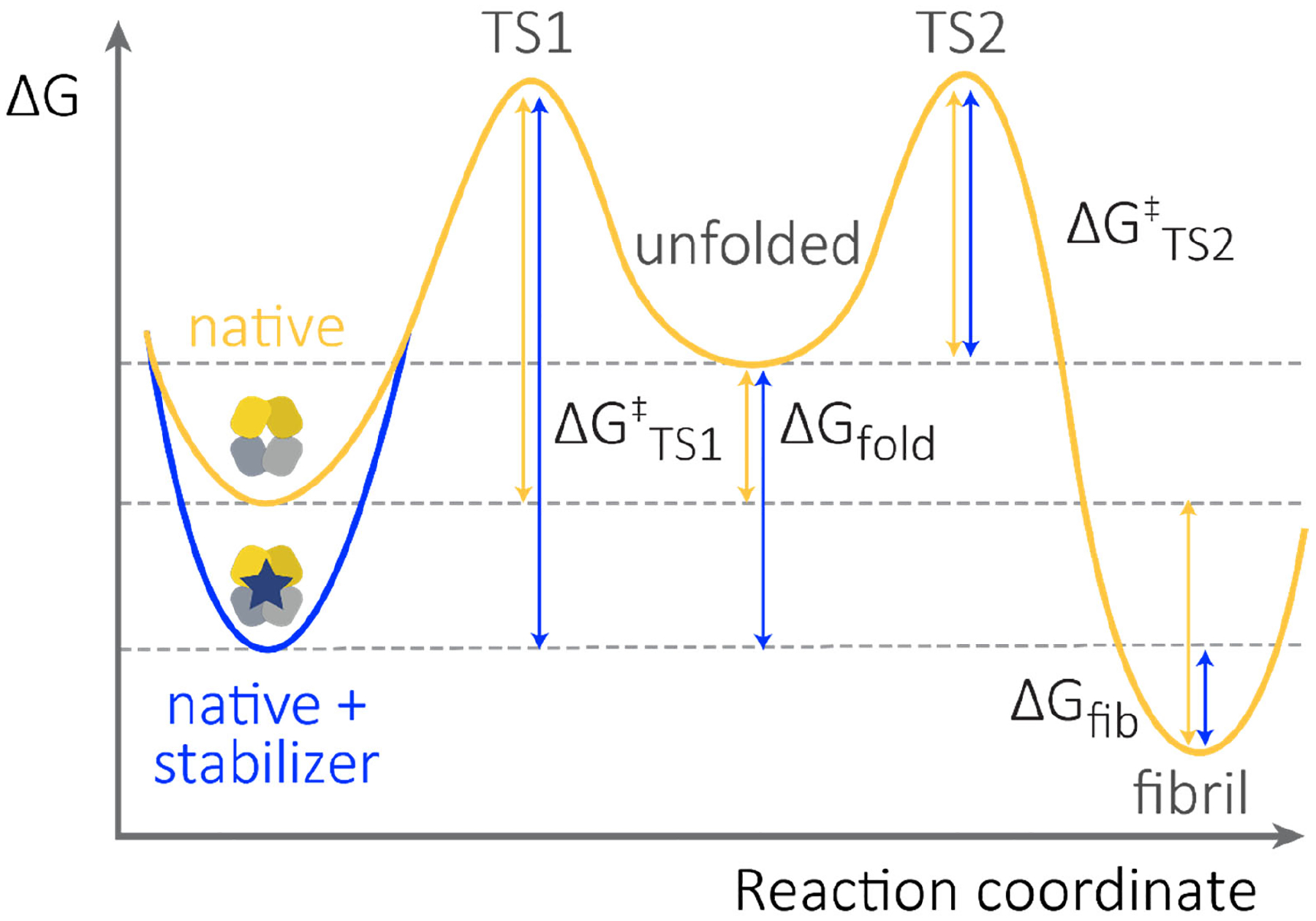
Small molecule stabilizers enhance the thermodynamic and kinetic stability of FL LCs. Schematic free energy diagram showing the relative stabilities of LC species. Energy differences are not shown to scale. Fibril formation occurs due to self-association of non-native states and therefore requires unfolding from the native state. Note that the stability of the fibrils (ΔG_fib_) and the energy barrier to self-association (ΔG^‡^_TS2_) are concentration dependent. Thus, fibrils are favored at high non-native LC concentrations. Binding of a small molecule stabilizer will shift the equilibrium defined by ΔG_fib_ and ΔG_fold_ towards the native state (blue arrows), however the rate of this process is likely to be very slow. Therefore, small molecule kinetic stabilization—which increases the energy barrier for unfolding, ΔG^‡^_TS1_—is likely to be the most important attribute for a successful FL LC kinetic stabilizer drug candidate.

Substantial biophysical evidence demonstrates that non-amyloidogenic FL LCs are kinetically stable, meaning that they denature relatively slowly. Low levels of kinetically stable FL LCs are secreted as a byproduct of antibody production and secretion. In contrast, FL LCs secreted from AL patient clonal plasma cells are kinetically less stable, i.e., they denature relatively quickly (Figure 3) [58]. These protein chemistry attributes are important because the small molecule stabilizers we are developing impose kinetic stabilization on the amyloidogenic FL LCs by binding of a stabilizer molecule to the native state selectively over the unfolding transition state (our stabilizers will also bind the kinetically stable FL LCs secreted as a byproduct of antibody production). The selective binding of the native state increases the free energy barrier for unfolding (ΔG^‡^_TS1_; Figure 3) and thereby reduces the rate of partial denaturation, aberrant proteolysis requiring partial denaturation, and LC aggregation. Pharmacologic kinetic stabilization could be particularly relevant in AL amyloidosis, where the LC half-life in the circulation is typically short, 2–4 h, because the LCs are removed by the kidneys. Therefore, the system likely never reaches equilibrium. Instead, the steady state concentration of non-native LCs is largely determined by the rate of unfolding governed by ΔG^‡^_TS1_. Pharmacological kinetic stabilization slows LC unfolding, increasing the likelihood that the amyloidogenic LCs will be excreted by the kidneys before unfolding or unfolding and misassembling–processes linked to organ toxicity.

## 4. Progress to Date: Structure-Based Small Molecule FL LC Stabilizer Discovery

To develop FL LC kinetic stabilizers as drug candidates for AL, we needed to start with molecules that could bind to the native state of FL LCs. In principle, high-affinity binding of antibodies or other proteins could stabilize FL LCs. However, orally bioavailable small molecule kinetic stabilizers have multiple advantages, including enhanced patient compliance and very low drug cost once the pharmacological kinetic stabilizer goes off patent. Therapeutic antibodies that have been investigated to clear amyloid do not bind to the native FL LCs [78]. Using a FL LC native state binding antibody is challenging because the high molecular weight of the antibody:LC complex could also prevent clearance of LCs via the kidneys. Moreover, the majority of LC molecules would need to be engaged in vivo, requiring a high therapeutic antibody concentration. Additionally, a stabilizing antibody would need to selectively bind only to free LCs, to prevent formation of poly-antibody immune complexes. Although these challenges are not insurmountable, we decided to focus on small molecule drugs instead of biologics. Our investigations of other small molecules that had previously been identified by the Deutsch and Eisenberg Labs as potential inhibitors of LC aggregation suggest that these molecules do not act as FL LC native state stabilizers [60,61,79]. Importantly, we did not observe FL LC stabilization by doxycycline, an antibiotic that has been proposed to inhibit amyloid formation and disrupt pre-formed amyloid fibrils. Doxycycline has shown encouraging results in model systems and clinical trials [80–82] but it is a pleiotropic molecule with several potential mechanisms of action and our data do not support the hypothesis that doxycycline stabilizes the native state of FL LCs.

We therefore turned to high throughput screening to identify small molecules that could stabilize the native fold of FL LCs. Protein denaturation is required for proteases to bind and cut proteins [31]. To measure the kinetic stabilization of amyloidogenic FL LCs imposed by small molecule binding in vitro, we designed an assay that could be used in a microplate format to detect protection of FL LCs from added proteases [60]. A recombinant amyloidogenic FL LC was labeled with a fluorescent dye. Release of fluorophore-labeled peptides by aberrant proteolysis was quantified by fluorescence polarization. From a library of over 650,000 small molecules, this so-called “protease-coupled fluorescence polarization” assay identified multiple small molecule stabilizer hits that reduced the decrease in fluorescence polarization. These stabilizers were validated using a series of counter screens. We excluded molecules that interfered with the fluorescence polarization readout or inhibited proteinase K, the challenge protease used in this assay. Surviving were small molecule FL LC stabilizer hits from five distinct structural families.

**Figure 4.**
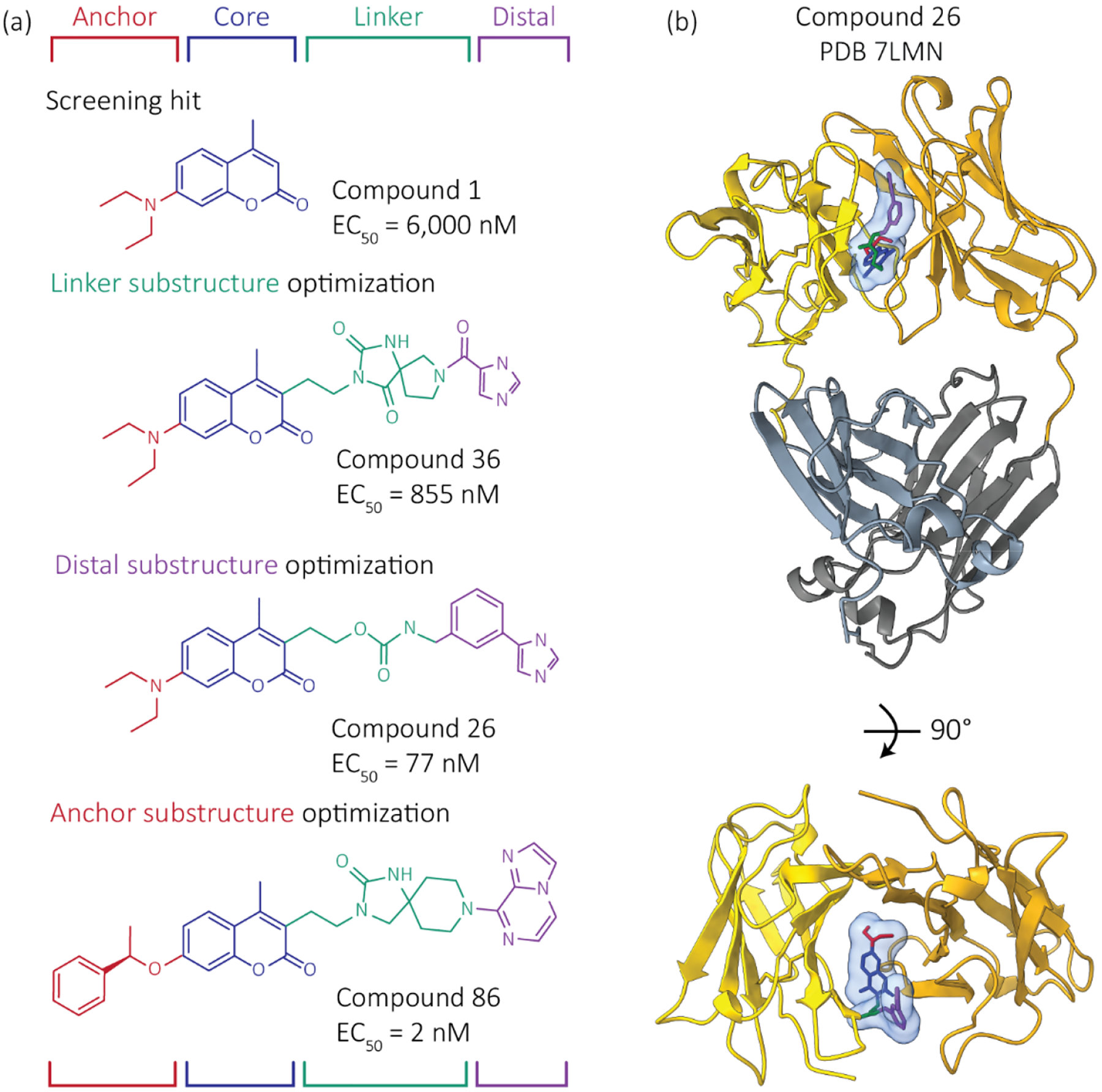
Development of more potent FL LC kinetic stabilizers. Starting from the structure of coumarin hit molecule **1** bound to a FL LC dimer, as well as the structures of other hit kinetic stabilizers bound to FL LC dimers, we used the principles of structure-based design to blueprint, dock and synthesize >500 novel molecules aimed at improving the affinity and selectivity of our kinetic stabilizers for FL LC dimers. Our modular approach optimized the indicated four regions of a kinetic stabilizer candidate somewhat independently, then we combined the best performing substructures. Colors represent regions of the kinetic stabilizers. (**a**) Structures and activities of example kinetic stabilizer molecules, numbered as in Yan et al. [83]. EC_50_ values describe the concentration of kinetic stabilizer necessary to protect LCs from proteolysis. (**b**) Crystal structure of Compound **26** bound to a FL LC dimer is shown. The C-regions are hidden in the bottom image for clarity.

NMR spectroscopy, competition binding experiments, computational modeling and X-ray crystallography consistently identified a small molecule binding site located between the two V-regions, i.e., at the variable domain-variable domain interface in the FL LC dimer (Figure 4) [60]. The binding site is comprised of residues that form the interface between the LC and its heavy chain (HC) partner in antibodies. Thus, the FL LC residues that bind the small molecule kinetic stabilizers are highly conserved amongst FL LCs. Significantly, the structure adopted by these residues in the FL LC homodimers is distinct from the HC:LC interface in an intact antibody. No binding was observed between hit molecule **1** and a human antibody Fab (a dimer of the FL LC and HC Vh-Ch1 region). It would have been difficult to identify the FL LC kinetic stabilizer binding site by computational methods because its conformation differs in the small molecule kinetic stabilizer-FL LC dimer complex versus the isolated FL LC dimer—the two variable domains move relative to each other, opening a cavity in which the kinetic stabilizer can bind.

Although the hits from the screen exhibited modest dissociation constants, ≈5 μM, they demonstrated that pharmacologic stabilization of FL LCs could be achieved by binding to this conserved variable domain-variable domain kinetic stabilizer binding site (Figure 4). With multiple structures from screening hits to guide our efforts, we were able to design new FL LC kinetic stabilizers that were more potent [83,84]. To date, we have designed and synthesized over 500 small molecules, the most potent of which exhibited single digit nanomolar EC_50_ values for FL LCs (Figure 4), data comparable to that exhibited by the FDA-approved TTR kinetic stabilizer tafamidis [85]. The increase in stabilizer binding affinity translates into increased protection of unstable FL LCs against added proteases, including isolated variable domains.

## 5. Potential Clinical Utility in AL

An efficacious, well-tolerated FL LC dimer small molecule kinetic stabilizer should substantially reduce soluble non-native LC levels that appear to drive pathology in AL. A FL LC small molecule kinetic stabilizer should also prevent newly secreted FL LCs from adding to existing amyloid present in tissue. These combined attributes are expected to benefit most if not all AL patients, particularly those who are very ill. Thus, we envision that pre-treatment of patients with cardiac involvement with a pharmacologic kinetic stabilizer will improve their overall health sufficiently to allow them to better tolerate cytotoxic anti-plasma cell eradication therapies.

Organ responses to therapy in AL correlate with deep hematological responses. However, not all patients reach or sustain such a response (Table 1), even in the era of targeted agents such as daratumumab [35]. Therefore, we envision FL LC kinetic stabilizers playing a major role in improving the organ response of patients whose clonal cells do not completely respond to therapy. In these patients, the low levels of destabilized FL LCs that continue to be secreted will be rendered unable to misfold and misassemble by kinetic stabilizer binding.

Although many patients now respond to current AL therapies, most remain at risk of relapse. The clonal plasma cells in AL are less proliferative than those that cause myeloma, so rapid relapse appears to be rare, although transition to overt myeloma is possible [86]. Instead, progression of symptoms appears to be driven by creation of new amyloid fibrils or growth of residual deposits. No well-defined criteria have been determined for relapse after hematologic response. This is partly because it is not possible to predict how existing amyloid deposits will respond to new soluble LCs. There is significant debate over the optimal way to treat patients whose amyloidogenic FL LC levels begin to increase after a period of remission, since the cytotoxic therapies are expensive, difficult to tolerate, and are prone to generate resistance [39,87,88]. We hypothesize that the concentration of non-native monoclonal LCs determines the risk of relapse or progressive loss of organ function. Pharmacologic FL LC kinetic stabilization could extend remission and allow patients to maintain their quality of life by substantially reducing these non-native LC levels, even if clonal plasma cell levels slowly increase over time.

Because LCs appear to play no role in plasma cell proliferation after secretion, we predict that mutations in LC genes that disrupt small molecule binding will not confer a selective advantage to the plasma cells producing them. Therefore, resistance to FL LC small molecule kinetic stabilizer therapy is unlikely to develop.

## 6. Considerations for Using FL LC Kinetic Stabilizers

Although FL LC dimers have no known function in vivo, the potential adverse effects of stabilizing these well-defined structures are not known. The circulation of stable LCs in asymptomatic monoclonal gammopathy or even in multiple myeloma indicates that the therapeutic window for stabilization should not be a problem. Clinical experience with TTR stabilizers is also encouraging in this regard. However, caution is warranted because animal models of AL do not recapitulate all features of the disease and only human clinical trials can reveal unexpected toxic mechanisms. The μM concentrations of a pharmacological FL LC kinetic stabilizer that would be required to stabilize all FL LC dimers in circulation means that a candidate would need to display an exemplary safety profile to be highly useful.

Light chains are cleared by the kidneys [89,90], and renal involvement occurs in two-thirds of AL patients [25]. Therefore, investigating the effect of LC stabilization on renal function will be important. Individuals with healthy kidney function are expected to metabolize stabilized LCs normally within tubular epithelial cells, assuming that renal blood flow and glomerular filtration are maintained at sufficient levels. For AL patients who have reduced renal function, LCs may be excreted intact as BJPs and not contribute further to pathology. However, it is possible that kinetic stabilizer-bound FL LCs will be proteolyzed and/or excreted more slowly by the kidney, leading to accumulation and kidney damage by non-amyloid mechanisms.

While preliminary results argue against this possibility [60], kinetic stabilizers of FL LCs could possibly reduce the folding and secretion of antibodies in healthy plasma cells by enabling more FL LC secretion by way of a pharmacologic chaperoning mechanism. In other words, if the FL LC kinetic stabilizer substantially enhanced the amount of FL LC dimer that is normally secreted along with antibodies, at the expense of antibody assembly and secretion, this could be problematic in normal plasma cells [91]. However, biosynthetic studies in myeloma cells producing intact IgG’s revealed relatively balanced synthesis of HC and LC (as would be the case in normal antibody producing plasma cells) and indicate that the binding of LC to HC is relatively efficient [92]. Similarly, the thermal stability of antibody Fabs is typically higher than that of homodimeric BJPs.

Kinetic stabilizers could render proteasome inhibitors less effective at killing the plasma cell clone, because pharmacologic stabilization of FL LCs within the endoplasmic reticulum of a plasma cell clone may reduce proteostatic stress, which could potentially reduce the sensitivity of plasma cells to bortezomib or analogous drugs [93].

These potential challenges are important to consider in any eventual clinical trial of FL LC kinetic stabilizers.

## 7. Conclusions and Perspective

Unfolding of FL LC dimers, and in some cases their subsequent aberrant proteolysis products, appears to be required for LC misassembly into a spectrum of soluble non-native structures that are responsible for organ toxicity in AL amyloidosis. Pharmacological stabilization of the FL LC dimer native state should suppress formation of toxic non-native LC species, thereby benefiting patients. The lower than desired rate of organ response after apparently successful clonal plasma cell eradication is envisioned to result from a small population of plasma cells that continue to secrete the amyloidogenic FL LC. We have pharmacologically stabilized sequence diverse FL LCs against unfolding or unfolding and proteolysis because binding utilizes highly conserved FL LC amino acid residues. The transthyretin kinetic stabilizer tafamidis was the first regulatory agency approved drug to show clinical benefit [13,94] in transthyretin amyloidosis, followed by demonstration of the efficacy of the TTR “silencing” drugs of Alnylam and Ionis [95,96]. In AL, the efficacy of FL LC lowering drugs was demonstrated first (i.e., clonal plasma cell eradicators) and it stands to reason that the FL LC kinetic stabilizers will also be effective. Now, TTR amyloidosis trials are underway to evaluate the added benefit to the patient of using a kinetic stabilizer and a TTR lowering agent, and we expect that, in a similar fashion, FL LC kinetic stabilizers will complement the existing clonal plasma cell eradication therapies for AL.

## Figures and Tables

**Table 1.** Summary of selected studies investigating the efficacy of different therapies in AL. In each study, organ responses were observed in only a subset of patients in whom a hematological response (HR) was observed, although deeper HRs are associated with higher rates of organ response. Median overall survival is shown as a range for autologous stem cell transplant, from no hematologic response to a complete response. When median overall survival was not reached, the maximum follow-up time is shown.

Study Reference	Treatment	Dates	Number of Patients	Patient Status	HR (CR)	Renal Response	Cardiac Response	Median Survival
Szalat et al. [36]	Autologous stem cell transplant	2002–15	206	ND	69% (28%)	54%	62%	3.7–14.5 yr
Kastritis et al. [41]	Melphalan + dexamethasone + bortezomib	2011–16	53	ND	79% (23%)	44%	38%	>50 mo
Kastritis et al. (control) [41]	Melphalan + dexamethasone	2011–16	56	ND	52% (20%)	43%	28%	34 mo
ANDROMEDA [35]	CyBorD + Daratumumab	2018–19	195	ND	91.8% (53.3%)	53.0%	41.5%	>20 mo
ANDROMEDA (control) [35]	CyBorD	2018–19	193	ND	76.7% (18.1%)	23.9%	22.2%	>20 mo
Sanchorawala et al. [42]	Daratumumab + dexamethasone	2017–19	22	RR	90% (41%)	67%	50%	>28 mo
Roussel et al. [43]	Daratumumab + dexamethasone	2018–19	40	RR	70% (15%)	31%	29%	>36 mo
Milani et al. [40]	Pomalidomide + dexamethasone	2009–18	153	RR	44% (3%)	20%	11% *	29 mo
TOURMALINE-AL1 [44]	Ixazomib + dexamethasone	2012–18	85	RR	53% (26%)	28%	18%	34.8 mo

CyBorD, cyclophosphamide, bortezomib and dexamethasone; ND, newly diagnosed; RR relapsed/refractory disease; CR, complete hematological response.

* Pomalidomide can elevate the NT-proBMP biomarker used to evaluate cardiac response [40], so cardiac responses may be higher in patients treated with this agent.
